# Petri Net and Probabilistic Model Checking Based Approach for the Modelling, Simulation and Verification of Internet Worm Propagation

**DOI:** 10.1371/journal.pone.0145690

**Published:** 2015-12-29

**Authors:** Misbah Razzaq, Jamil Ahmad

**Affiliations:** Department of Computational Sciences, Research Center for Modeling & Simulation (RCMS), National University of Sciences and Technology (NUST), Islamabad, Pakistan; Tianjin University of Technology, CHINA

## Abstract

Internet worms are analogous to biological viruses since they can infect a host and have the ability to propagate through a chosen medium. To prevent the spread of a worm or to grasp how to regulate a prevailing worm, compartmental models are commonly used as a means to examine and understand the patterns and mechanisms of a worm spread. However, one of the greatest challenge is to produce methods to verify and validate the behavioural properties of a compartmental model. This is why in this study we suggest a framework based on *Petri Nets* and *Model Checking* through which we can meticulously examine and validate these models. We investigate *Susceptible-Exposed-Infectious-Recovered (SEIR)* model and propose a new model *Susceptible-Exposed-Infectious-Recovered-Delayed-Quarantined (Susceptible/Recovered) (SEIDQR(S/I))* along with hybrid quarantine strategy, which is then constructed and analysed using *Stochastic Petri Nets* and *Continuous Time Markov Chain*. The analysis shows that the hybrid quarantine strategy is extremely effective in reducing the risk of propagating the worm. Through *Model Checking*, we gained insight into the functionality of compartmental models. *Model Checking* results validate simulation ones well, which fully support the proposed framework.

## 1 Introduction

### 1.1 Internet Worm

Since the discovery of the first internet worm in 1988 viz. *Morris* Worm, systems running on networks are more prone to digital threats [1]. The safety and security of the internet have been compromised particularly by worms that exploit zero hour vulnerabilities. The sudden advancement of computer technologies and network applications have become a potential haven for malicious software programs. The propagation behaviour of worms on internet can somewhat be correlated with biological diseases [2, 3]. Some notable worms including *Code Red* and *Nimda* attacked hundreds of thousands of computers in 2001 [4, 5]. *Blaster* worm (2003) employed sequential scanning to find its targets [6]. *SQL Slammer* infected more than ninety percent vulnerable computers within 10 minutes in 2003 [7]. Witty worm was the first wide spreading worm that damaged infected hosts [8]. *Storm* worm infected thousands of computers in 2007 [9]. *Conficker* was detected in November 2008 is the largest known worm since *SQL Slammer* [10]. Worm propagation speed is directly proportional to the bandwidth and automatic mitigation is the only solution to stop their propagation because manual countermeasures are very slow. Network intrusion detection techniques are used for this purpose and can be divided into two categories: signature based and anomaly based. Every technique has its own pros and cons. Signature based techniques cannot detect unknown worms while anomaly based techniques have high false positive rates [11]. Recently, Entropy measure methods have been proposed [12–15] to study the robustness and complexity of the network.

### 1.2 Existing Models

Worm propagation models are used to understand propagation behaviour in order to develop appropriate defence mechanisms against future attacks [16]. A variety of worm propagation models have been proposed to study the worm’s spread and the effectiveness of defensive strategies. Most of the these models [5, 17, 18] are based on the Kermack-Mckendrick model. By the use of worm propagation models, Anderson and May have thoroughly explained the behavioural nature of biological diseases and parasites that can lead to the propagation of infectious diseases in human population [19]. By applying the same method, via using the epidemiological models for disease propagation we can monitor and study the behaviour of worms throughout a network [20]. The Susceptible-Exposed-Infectious-Recovered-Susceptible (SEIRS) model presented by Mishra and Saina have latent and temporary periods that identify the propagation of a common worm [21]. Based on the Susceptible-Exposed-Infectious-Recovered (SEIR) model, Dong et al. proposed a computer virus propagation model and studied the dynamical behaviour including local asymptotical stability and local Hopf bifurcation of a computer virus model using time delay as a bifurcating parameter [22]. L.-X. Yang and X. Yang, examined the dynamics of the virus propagation, once infected systems are running on the network with positive chance [23]. Under human intervention Gan et al. examined the computer virus propagation behaviour [24]. Ren et al. gave a new computer virus dispersion model and studied different dynamic behaviour of the model [25]. Quarantine is common and an effective way of containing the worms [7]. The use of quarantine containment method has produced some extraordinary results, successfully regulating diseases [7, 19–26]. Wang et al. combined both a dynamic quarantine strategy and a vaccination in an epidemic model and referred this new model as SEIQV model [27]. Zou et al. proposed a new model with dynamic quarantine strategy based on two-factor model [7]. Xia et al. proposed a new model with direct immunization [28]. Xia et al. examined the SIRS model to determine the effect of non uniform transmission [29]. Xia et al. proposed a new epidemic model with infection delay and propagation vector [30]. Sanz et al. proposed a new framework to study the dynamics of concurrent diseases [31]. Wang et al. comprehensively reviewed the latest work on the spatial meta population [32]. Cattuto et al. presented a scalable framework to monitor the social interaction and study the dynamics of face to face interaction [33]. Zhang et al. studied the contact network from temporal point of view [34]. Driessche et al. presented a SIRI model for a disease with relapse [35]. Driessche et al. developed a SEIRI compartmental model [35].

### 1.3 Our Contribution

According to our knowledge, previous studies have not taken into account the effect of time delay in detecting worm and applying countermeasures on susceptible, exposed and infectious hosts. To cope with this delay, delayed state is introduced in the proposed model. To make our results more realistic, we have introduced a transition from recovered to susceptible and infected state because a network can never be worm free as there is always a chance of re-infection. Our proposed model is based on the hybrid intrusion detection, which combine features of both signature based and anomaly based techniques. According to the above description, we have presented a Susceptible-Exposed-Infectious-Recovered-Delayed-Quarantined (Susceptible/Recovered) “SEIDQR(S/I)” worm propagation model by modifying *Susceptible-Exposed-Infectious-Recovered (SEIR*), to study the behaviour of worm’s spread and to analyse the effectiveness of a quarantine strategy through *Model Checking*. This work presents the framework for studying worm’s propagation and validating the compartmental models. We have used *Petri Nets* and Model Checking to look closely at the behaviour of the model. *Stochastic Petri Net (SPN)* of the *SEIDQR(S/I)* is constructed and simulated in *Snoopy* Tool. *Continuous Time Markov Chain (CTMC)* of the model is generated in *PRISM* model checker to formally verify the behavioural properties of the model. The *Charlie* tool is used to analyse qualitative properties of the *SPN*. Through *SPN* we have created many experiments and have studied the impact of different parameters and classes on the system. According to our knowledge, framework based on *Petri Nets* and *Model Checking* has not been previously proposed for this purpose. Our paper presents the first approach in this direction. This framework offers promising advantages in terms of qualitative and quantitative analysis of compartmental models.

### 1.4 Structure of the Paper

The rest of the paper has been structured in the following manner: Section 2 gives an overview of the proposed *SEIDQR(S/I)* model and illustrates the *SPN* and *CTMC* of the proposed model. In section 3, we show the simulations using *Snoopy* tool, quantitative analysis using *PRISM* model checker and qualitative analysis using *Charlie* tool. It also shows the analysis of the hybrid quarantine strategy through *PRISM* model checker. We discuss the methodology and results in section 4. Conclusions are drawn on the basis of results in section 5. A list of abbreviations (S1 Table) is provided at the end of this article.

## 2 Methods

**Fig 1:**
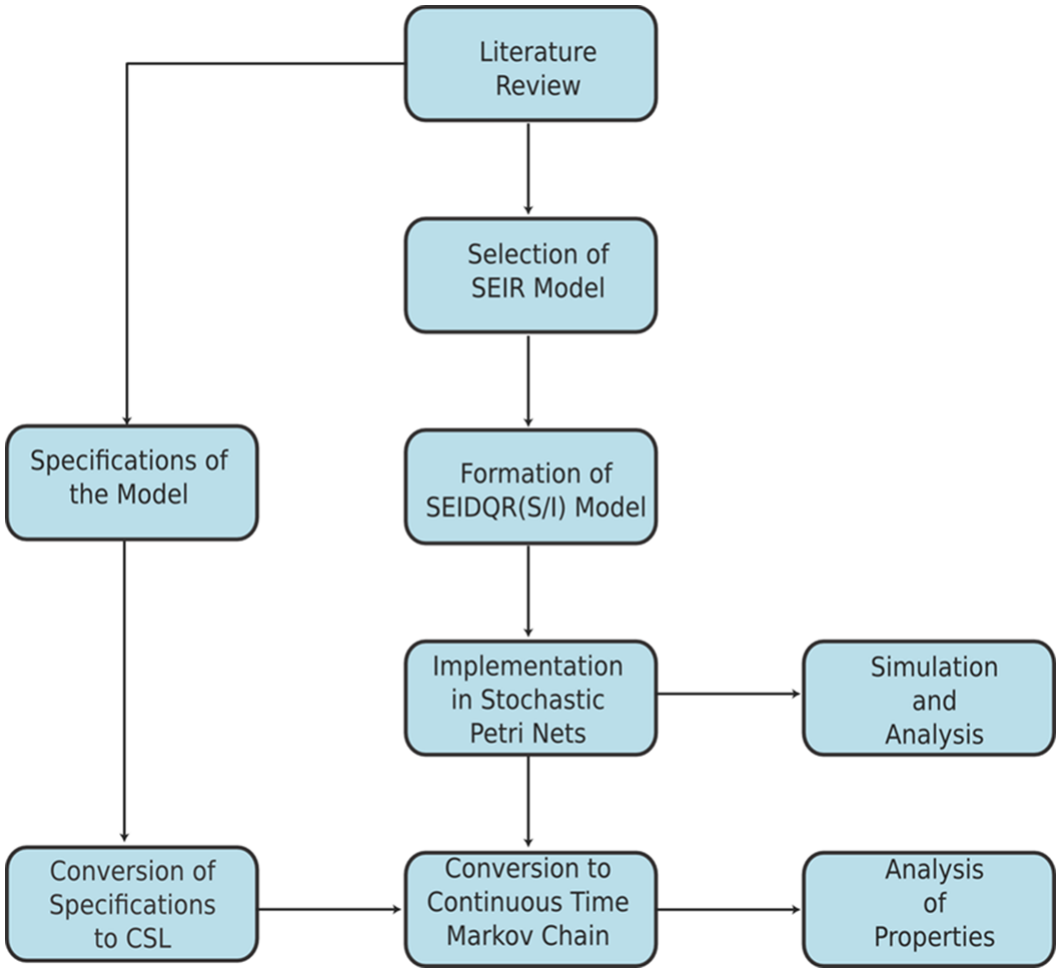
Flow chart of the Proposed Framework. After reviewing literature, *SEIR* model is selected and a new *SEIDQR(S/I)* is proposed by modifying SEIR model. *SPN* of the proposed model is constructed and analysed in *Snoopy* and *Charlie*, after which the system is converted to *CTMC* and specifications are encoded in CSL for quantitative analysis in *PRISM* model checker.

This research is an exploratory study on how worms propagate and how their parameters have an effect on their propagation speed. Worm propagation models are used to understand propagation behaviour in order to develop appropriate defence mechanisms against future attacks. Current worm propagation modelling techniques are based on complex differential equations. *Petri Nets* formalism can be used to develop worm propagation models with less complexity [36]. What is advantageous is that it is not such a laborious task to develop worm propagation models using *Petri Nets*. We can also look closely at the behaviour of the model through *Model Checking* and this offers promising advantages in terms of qualitative and quantitative analysis. Fig 1 presents the structure of the proposed framework.

### 2.1 Model Formulation

Taking into account the worms having exploited zero day vulnerabilities, the host could not be immunized by the usually effective and reliable safety patches. Susceptible hosts initially face an incubation period (exposed) prior to becoming infectious, for this reason people may try placing countermeasures as a precautionary attempt to immunize the exposed and infectious host. However, this can be time consuming and potentially aggravate the harm caused by worms if they are unknown. Due to the existence of the delays, the infectious hosts are put through a temporary state (delayed) before quarantining and recovery. We introduced delayed stage prior to the quarantined stage in order to compensate for the delays experienced by the hosts.

It is important to note that there is no permanent immunity in the real network. The only immunity a node achieves in a real network is temporary, for this reason we must observe the real phenomena of possible re-infection. In order to address this issue, a node reverts to the susceptible and infectious compartment again in the proposed model.

**Fig 2:**
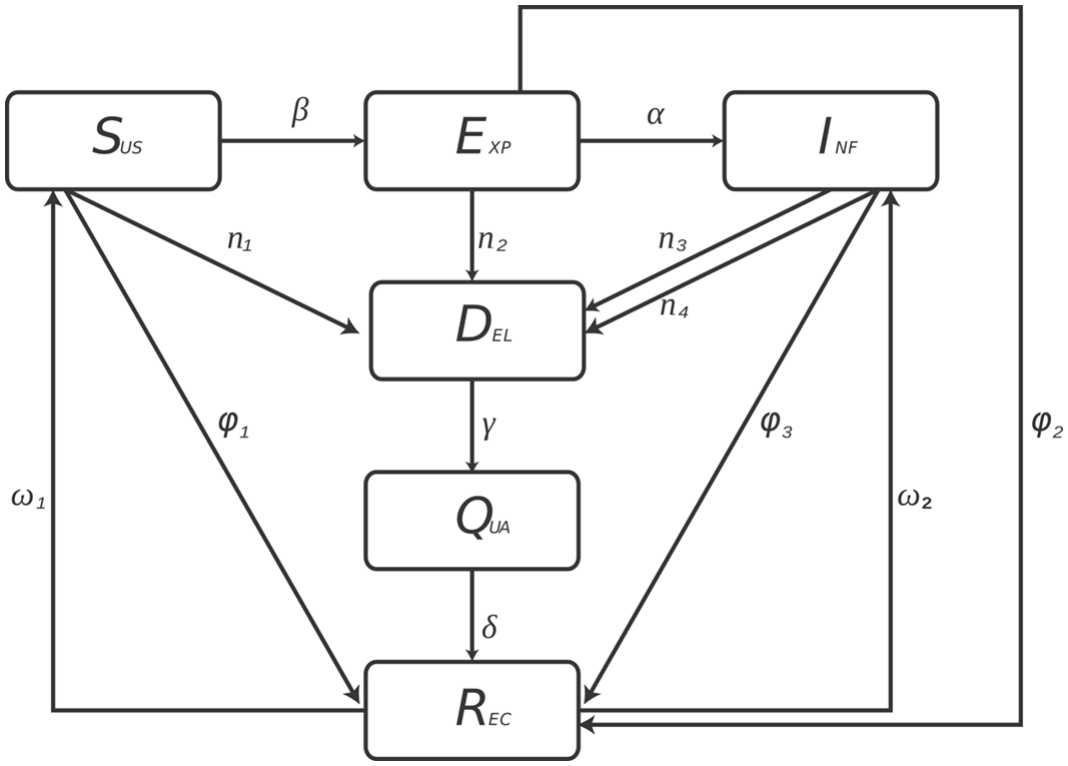
The states and the transitions of *SEIDQR(S/I)* model. The rectangles represent the compartments and the arrows represent the movement of hosts from one compartment to another. The labels on the rectangles indicate the type of compartment i.e. susceptible, exposed, infectious, delayed, quarantined and recovered. The labels on the arrows indicate the rate of transmission of hosts from one compartment to another.

Quarantine strategy is dependent on two types of intrusion detection systems, which can be categorized as misuse and anomaly intrusion detection. They both have their pros and cons but misuse intrusion detection systems recognizes the attack behaviour of potential threats with its broad database constructed of known attack behavioural patterns. This is beneficial to an extent but with regards to unknown worms variants, it fails to recognize them as a threat by not having the data of their behavioural patterns. On the contrary, anomaly detection systems are able to recognize abnormal behavioural patterns which help in the detection of unknown worms and their variants. The quarantine strategy proposed in this study is based on both, anomaly and misuse intrusion detection system. Although, anomaly detection technique can detect unknown worms, it is accompanied by false-positive rates and because of that two transitions have been added with rates *n*_1_ and *n*_2_ from susceptible and exposed classes (See Fig 2).

According to the above description, we have proposed a *SEIDQR(S/I)*) model with hybrid quarantine strategy. Presumably, the host will be in one of the following compartments: Susceptible Compartment (*S*_*US*_), Exposed Compartment (*E*_*XP*_), Infectious Compartment (*I*_*NF*_), Delayed Compartment (*D*_*EL*_), Quarantined Compartment (*Q*_*UA*_) and Recovered Compartment (*R*_*EC*_) with initial condition *N* = *S*_*US*_(*t*_0_) + *E*_*XP*_(*t*_0_) + *I*_*NF*_(*t*_0_) + *D*_*EL*_(*t*_0_) + *Q*_*UA*_(*t*_0_) + *R*_*EC*_(*t*_0_). Here, *S*_*US*_(*t*_0_), *E*_*XP*_(*t*_0_), *I*_*NF*_(*t*_0_), *D*_*EL*_(*t*_0_), *Q*_*UA*_(*t*_0_) and *R*_*EC*_(*t*_0_) represents the number of susceptible nodes, exposed nodes, infectious nodes, delayed nodes, quarantined nodes and recovered nodes at time instant *t*_0_. Fig 2 represents the proposed *SEIDQR(S/I)* model. *β* is the rate of transfer from susceptible to exposed compartment. *α* is the rate of transfer from exposed to infectious compartment. Parameters used in the proposed model are listed in Table 1.

**Table 1:** Notations and Explanation.

Notation	Explanation
N	Total size of population
*S*_*US*_(*t*)	Number of susceptible nodes at time instant *t*
*E*_*XP*_(*t*)	Number of exposed nodes at time instant *t*
*I*_*NF*_(*t*)	Number of infectious nodes at time instant *t*
*D*_*EL*_(*t*)	Number of delayed nodes at time instant *t*
*Q*_*UA*_(*t*)	Number of quarantined nodes at time instant *t*
*R*_*EC*_(*t*)	Number of recovered nodes at time instant *t*
*β*	Rate of transfer from susceptible to exposed compartment
*α*	Rate of transfer from exposed to infectious compartment
*γ*	Quarantined rate of delayed nodes
*δ*	Recovery rate of quarantined nodes
*n*_1_	Delayed rate of susceptible nodes
*n*_2_	Delayed rate of exposed nodes
*n*_3_	Delayed rate of infectious nodes with anomaly detection
*n*_4_	Delayed rate of infectious nodes with signature based detection
*φ*_1_	Rate of transfer from susceptible to recovered compartment
*φ*_2_	Rate of transfer from exposed to recovered compartment
*φ*_3_	Rate of transfer from infectious to recovered compartment
*ω*_1_	Rate of transfer from recovered to susceptible compartment
*ω*_2_	Rate of transfer from recovered compartment to infectious compartment

### 2.2 Petri Nets

*Petri Nets* theory was originated from Carl Adam’s Publications in 1962. *Petri Nets* are a powerful graphical and mathematical tool used to describe systems and their behaviour. We can analyse behavioural properties of the system using *Petri Nets* with relative ease [37].

*Petri Net* is a bipartite graph consisting of places in one partite and transitions in another. Places and transitions are connected through directed edges. If there is a directed arc from place *p* to a transition *t* then *p* is the input place of the transition *t*. If there is a directed arc from transition *t* to a place *p* then *p* is the output place of the transition *t*. If every input place of a transition contains token then transition is fire-able. Firing of a transition consist of removing a token from every input place and adding a token to every output place of a transition. Firing of transition results into the new marking of the *Petri Net*, for example to reach marking *M*_*z*_ from *M*_*y*_, there exist a firing sequence *S* [38].

#### 2.2.1 Standard Petri Net

**Fig 3:**
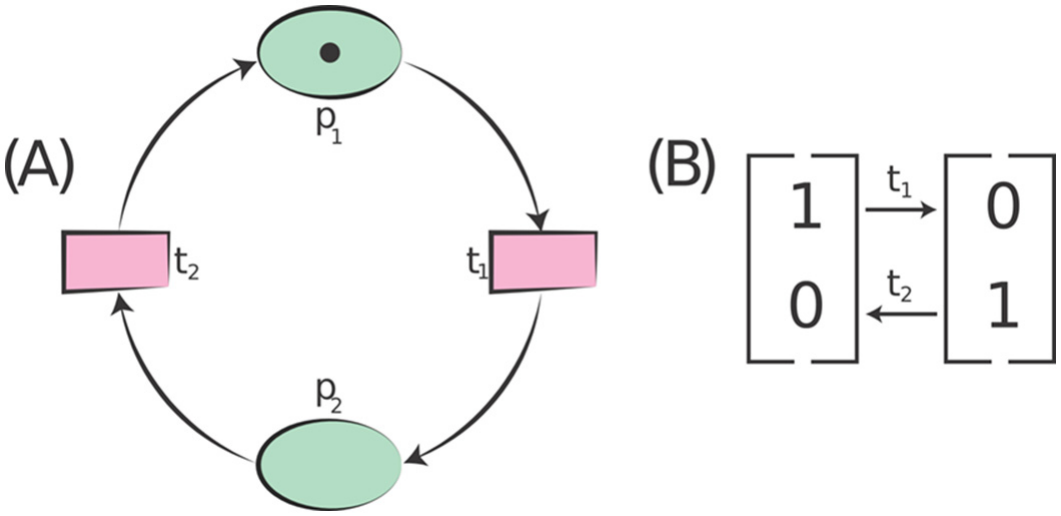
Example of a *Standard Petri Net*. (A) A *Petri Net* consists of a set of places {*p*_1_, *p*_2_}, set of transitions {*t*_1_, *t*_2_} and an initial marking *M*_0_ consisting of one token in place *p*_1_. In this example, the weight of the arcs are not specified so every arc weighs 1. The enabling degree of a transition is determined by number of times a transition can be fired without depositing a token again to the input place of a transition through self-loop. In case of above example *t*_1_ is 1 enabled and *t*_2_ is 0 enabled from the initial marking *M*_0_. (B) The reachability graph obtained from initial marking *M*_0_ of the *Petri Net*. A reachability graph consist of set of places which can be reached from *M*_0_ and arcs which are labelled with enabled transitions. This graph shows one cycle: (1, 0) → (0, 1) → (1, 0) and contains no deadlock. To reach marking *M*_1_ = (0, 1) from the marking *M*_0_ = (1, 0), a firing sequence *S* consist of a transition *t*_1_ once and transition *t*_2_ zero time.

“A *Standard Petri Net* [37] is defined by 5 − *tuple* ⟨*P*, *T*, *F*, *W*, *M*_0_⟩ where;

- *P* is a finite set of places {*p*_1_, *p*_2_, …, *p*_*m*_}
- *T* is a finite set of transitions {*t*_1_, *t*_2_, …, *t*_*m*_}
- *F* ⊆ (*P* × *T*) ∪ (*T* × *P*) is a set of arcs
- *W* is a weight function of arcs
- *M*_0_: is initial marking *P* → {0, 1, 2, …}

where *P* ∩ *T* = ∅ and *P* ∪ *T* ≠ ∅”. The example in Fig 3 illustrates the definition of the *Standard Petri Net*.

#### 2.2.2 Stochastic Petri Net

“A *Stochastic Petri Net (SPN)* [39] is defined by 6 − *tuple* ⟨*P*, *T*, *F*, *W*, *M*_0_, Ω⟩ where *P*, *T*, *F*, *W*, *M*_0_ are same as described in the definition of the *Standard Petri Net* and *Ω* represents the function $\Omega :T\to R_{\geq 0}$ which assigns rate to the transition *t* ∈ *T* according to the negative exponential distribution function”. The evolution of Stochastic *Petri Net* is described by a *Continuous Time Markov Chain (CTMC)* and a state of a *CTMC* represents the one marking of the *Petri Net*. In other words, *CTMC* represents the reachability graph of the *Petri Net* [39].

**Fig 4:**
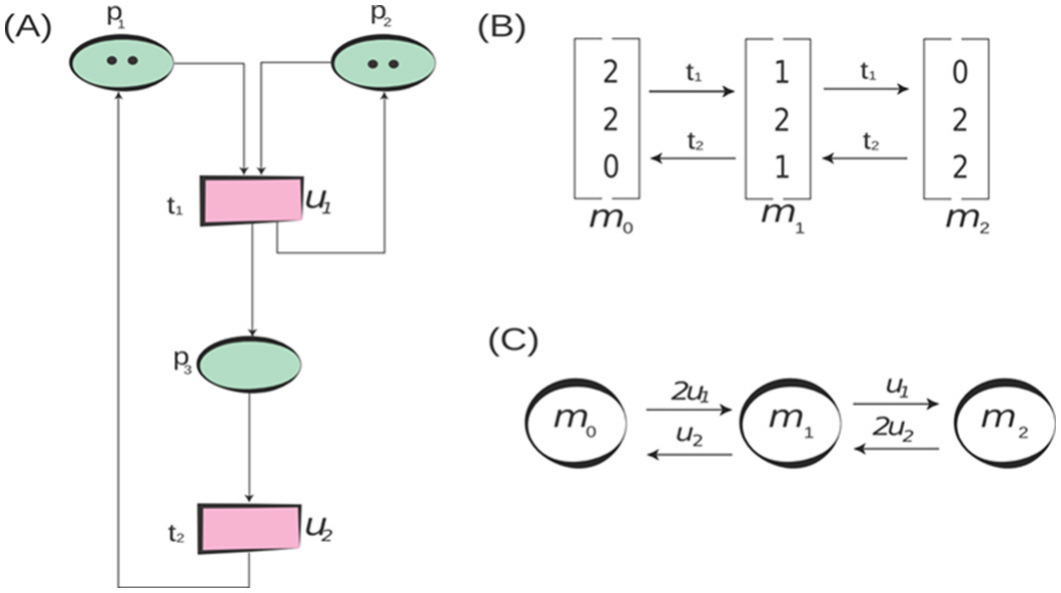
Example of a *Stochastic Petri Net*. (A) A *SPN* consists of a set of places {*p*_1_, *p*_2_, *p*_3_}, set of transitions {*t*_1_, *t*_2_}, rates *μ*_1_, *μ*_2_ and an initial marking *M*_0_ = (2, 2, 0). In case of this example *t*_1_ is 2 enabled and *t*_2_ is 0 enabled from the initial marking *M*_0_. (B) The reachability graph obtained from initial marking *M*_0_ of the *Petri Net*. (C) The *Markov Chain* obtained from the reachability graph in (B). Every reachable marking of the *SPN* is associated with a state of the *Markov Chain* and a transition between states is labelled with the product of the enabling degree and rate.

The example in Fig 4 illustrates the definition of the *Stochastic Petri Net*. There are several behavioural properties of *Petri Nets* [37, 38, 40] and some of these are described below:

- Reachability: This property is used to study dynamic properties of the system. A marking *M*_*k*_ is reachable from an initial marking *M*_0_ if there exists a firing sequence from *M*_0_ to *M*_*k*_.
- Liveness: A live *Petri Net* is a deadlock free *Petri Net* and from any marking, there exists a firing sequence which contain all transitions.
- Reversibility: This property ensures that there will always be a way back to the initial marking *M*_0_ from all reachable markings commencing from *M*_0_.

#### 2.2.3 SPN of the Proposed model

We have presented an approach through which we can model network epidemiological systems through *Petri Nets* with relative ease. *Petri Net* modelling of the real system is sometimes called Condition-Event net. *Petri Net* places are used to identify conditions of the system and transitions represent the flow from one condition to another. An event can only occur if all the conditions are satisfied i.e., input places are marked with sufficient tokens.

**Fig 5:**
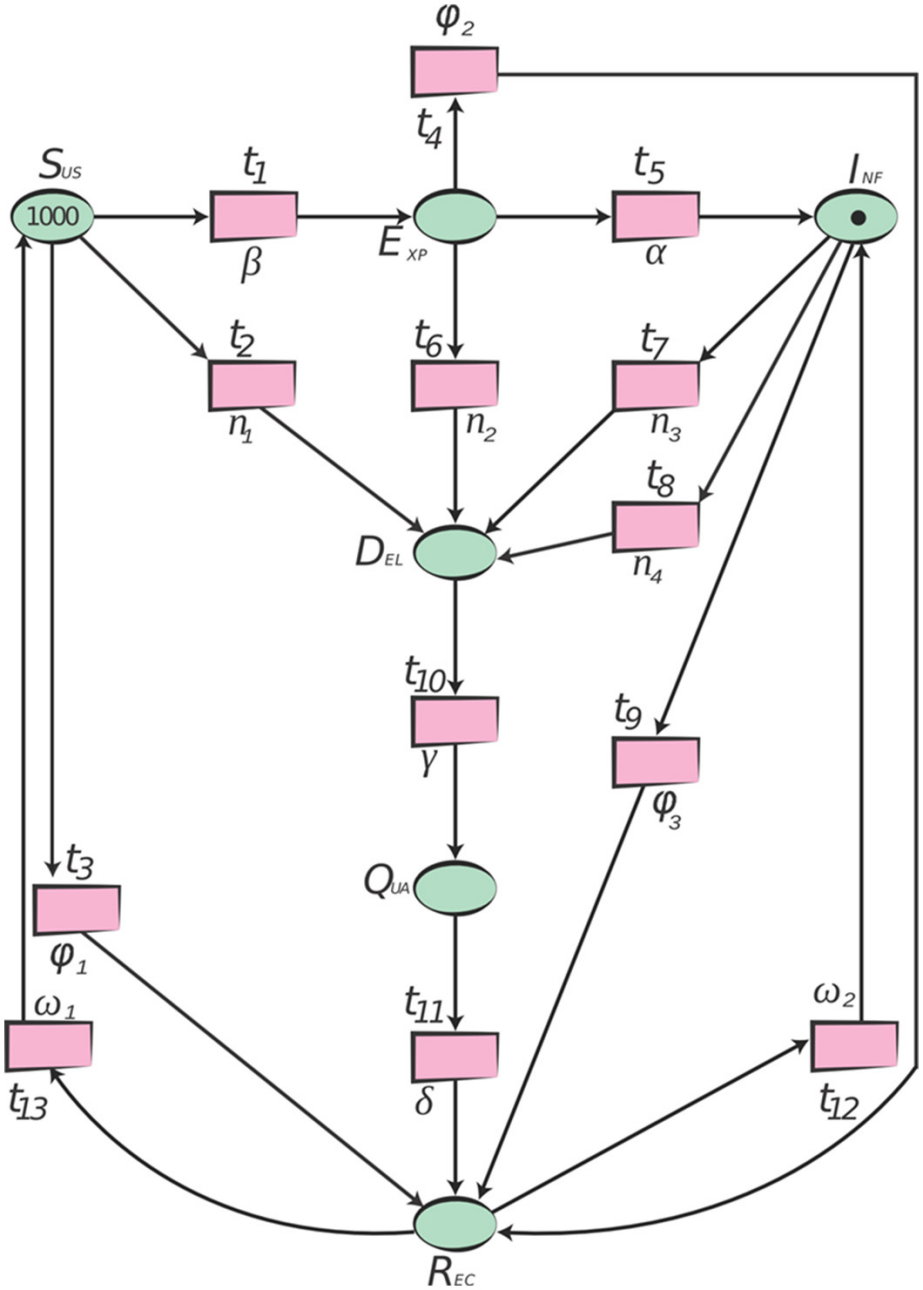
The *SPN* of the Proposed model. The *SPN* of the proposed model consists of a set of places *P* = {*S*_*US*_, *E*_*XP*_, *I*_*NF*_, *D*_*EL*_, *Q*_*UA*_, *R*_*EC*_} and set of transitions *T* = {*t*_1_, *t*_2_, *t*_3_, *t*_4_, *t*_5_, *t*_6_, *t*_7_, *t*_8_, *t*_9_, *t*_10_, *t*_11_, *t*_12_, *t*_13_} and initial marking *M*_0_ = (1000, 0, 1, 0, 0, 0).

We are using *SPN* to model our proposed system because we can easily generate *CTMC* though *SPN* as *SPNs* are isomorphic to *CTMC* [39]. To model an *SEIDQR(S/I)* as a *SPN*, we need to represent the host population which consist of different compartments. For this purpose, places are used to represent the states or compartments of the system i.e. susceptible, exposed, infectious, delayed, quarantined and recovered. Hosts are represented by the tokens and dynamic part of *SEIDQR(S/I)* is modelled by transitions labelled as *t*_1_, *t*_2_, *t*_3_, *t*_4_, *t*_5_, *t*_6_, *t*_7_, *t*_8_, *t*_9_, *t*_10_, *t*_11_, *t*_12_, *t*_13_ and these transitions originate the flow of hosts from one state to another with specified rate. Fig 5 illustrates the *SPN* of the proposed model.

### 2.3 Model Checking

**Fig 6:**
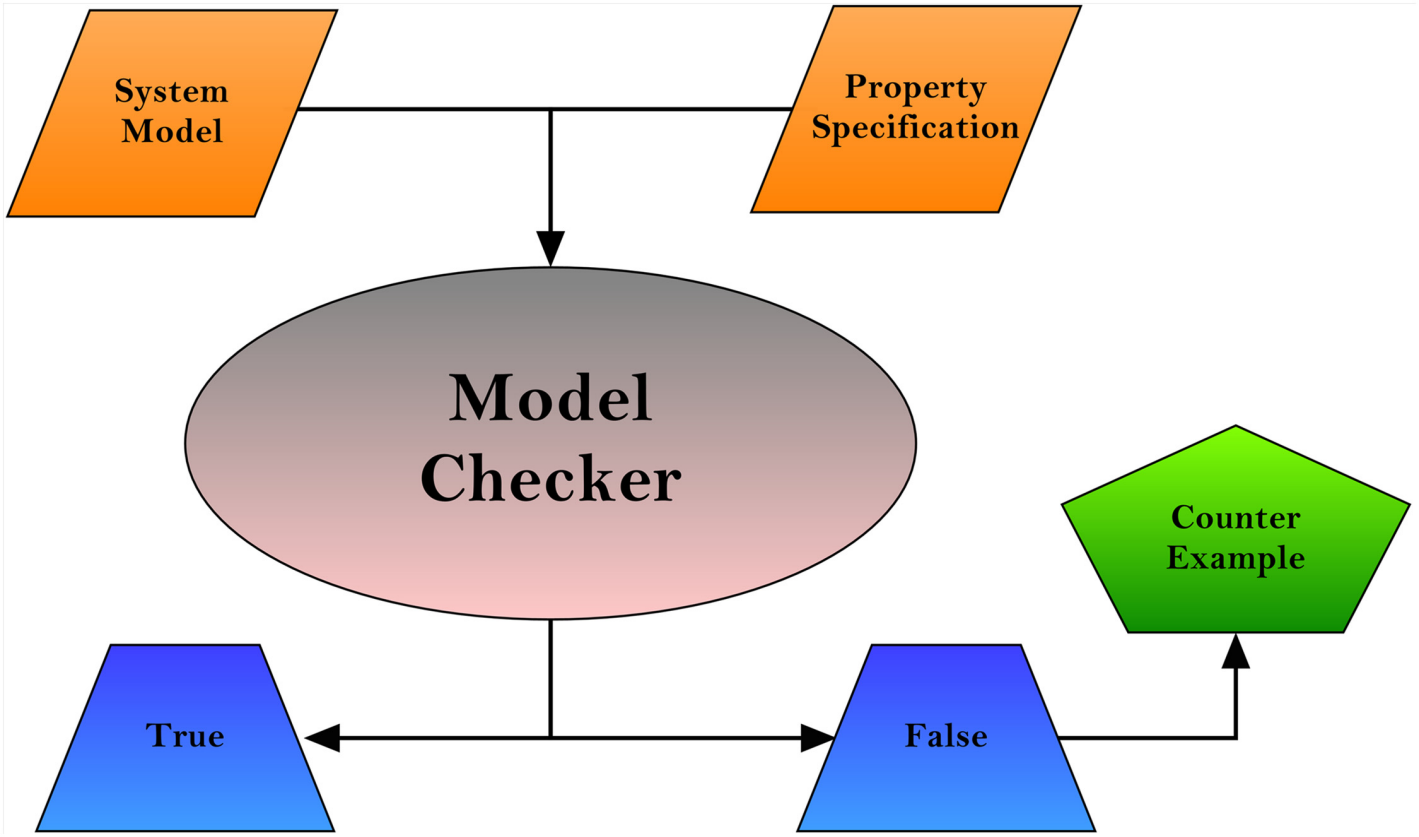
*Model Checking* Process. Model checker takes the system model and property specification as input and generates two types of output: (1) true which means property is satisfied (2) false with counter example which means property is not satisfied.

Testing or simulation-based system analysis techniques are not effective as compared to automatic model-based verification approaches [41]. Model checking, in particular, is a more powerful tool capable of exploring a whole state space. [42]. A model checker is used to examine whether the model of a system which is specified in some modelling formalism such as *Petri Nets*, meets the requirements of a system which are usually encoded in some temporal logic such as CTL (Computation Tree Logic) or PCTL (Probabilistic Computation Tree Logic) [43]. Fig 6 explains the *Model Checking* process.

#### 2.3.1 Probabilistic Model Checking

Probabilistic Model Checking is a variant of a formal verification method and is used for analysing and modelling a system which shows stochastic behaviours. There are two types of outputs of a probabilistic model checker: (1) ‘True’ signifying that the property is satisfied or ‘False’ signifying the property is dissatisfied. (2) ‘Numerical Number’ indicating the probability or expected time [44]. The *Model Checking* approach used in this study is probabilistic which is based on *CSL*. *PRISM* model checker is used for this purpose because *PRISM* supports *CTMCs* with *Continuous Stochastic Logic (CSL)*.

#### 2.3.2 Continuous Stochastic Logic

The temporal logic used in *CTMCs* is *CSL* which is based on both CTL and PCTL [45, 46]. It can specify path based, reward based as well as traditional properties [44]. The syntax of *CSL* is as follows:

$$\begin{array}{c} \phi ::=\;true\;|\;a\;|\;\neg \phi \;|\;\phi_{1}\wedge \phi_{2}\;|\;(P_{∼p}\left[\phi_{1}\cup^{I}\phi_{2}\right]\;\left|\;S_{∼p}\left[\phi \right]\;\right|\;R_{∼r}\left[F\phi \right] \end{array}$$

Where *a* represents the atomic proposition, ∼ ∈ {>, ≥, <, ≤}, *p* ∈ [0, 1], *I* is an interval of $R_{\geq 0}$ and $r\in R_{\geq 0}$.

*CSL* formulae are evaluated over the *CTMC* states. A formula *s* ⊨ *ϕ* indicates that *ϕ* is true in state *s* of *CTMC* model. *CSL* contains all standard operators from propositional logic: true (all states satisfy); atomic propositions (true in all states labelled with *a*); negation (¬*ϕ* is true in all states in which *ϕ* does not hold) and conjunction (*ϕ*_1_ ∧ *ϕ*_2_) (will be true in a state in which both *ϕ*_1_ and *ϕ*_2_ hold). We can drive other standard operators like disjunction and implication from these standard operators.

Furthermore, *CSL* includes probabilistic operators *P* and *S*. Both probabilistic operators include probability bound ∼*p*. We write *P*_∼*p*_[*Ψ*] to indicate the *CSL* formula *Ψ* is true in a state *s* if the probability of *CSL* path formula meets the bound ∼*p*. The *S* operator is used to verify the steady state behaviour of the *CTMC* in long run.

*CSL* also has another operator *R* to calculate those properties which require expected value of reward. *R*_∼*r*_[*Fϕ*] is used to calculate the accumulated value of the expected reward before a state is reached where *ϕ* is satisfied. [44, 47].

#### 2.3.3 CTMC of the Proposed Model

We have chosen a *Probabilistic Model Checking* approach because it provides both probabilistic analysis and conventional reachability. Through *Probabilistic Model Checking*, we will be able to accumulate accurate answers as compared to approximate solutions obtained through simulation. State space explosion is the only drawback of *Probabilistic Model Checking* [41].

We initiated the model definition, writing keyword *ctmc* in the beginning, to explicitly mention the type of probabilistic model used in this study. Then we mentioned the sequence of possible values upheld by state variables *S*_*US*_, *E*_*XP*_, *I*_*NF*_, *D*_*EL*_, *Q*_*UA*_ and *R*_*EC*_. In our case, we choose to let them vary between 0 and upper-limit labelled as Max. It is not necessary to specify upper-limits using parameters, but it helps to maintain the clarity of the model. Then, we labelled the module enclosing the transition rules as *SEIDQRSI*. After mentioning state variable names and defining their range within square brackets, keyword *init* is used to define the initial value of the variable. Then, we gave the definition of transition rules *β*, *α*, *γ*, *δ*, *n*_1_, *n*_2_, *n*_3_, *n*_4_, *φ*_1_, *φ*_2_, *φ*_3_, *ω*_1_, *ω*_2_. The list of transition rules are separated from the list of conditions using a symbol −>. We end the module by writing the keyword *endmodule*. We specified a reward structure by writing a reward rule between keywords *rewards* and *endrewards*.

We encoded behavioural properties in *CSL* in order to verify against the proposed model. Some of these behavioural properties are listed below:

- Expected number of hosts at any time instant *t*.
- Probability of reaching the maximum number of infectious hosts
- Invariance principle

*CTMC* of the Proposed Model is given in the supplementary file (S1 File).

## 3 Results

In this section, first we present the simulation results obtained through the *Snoopy* tool and then the verification results obtained through *PRISM* and *Charlie* tools.

### 3.1 Simulation Using Snoopy

Here we present the simulation results of the *Snoopy* tool. Initial values of the network are: *N* = 1001, *S*_*US*_(0) = 1000, *E*_*XP*_(0) = 0, *I*_*NF*_(0) = 1, *D*_*EL*_(0) = 0, *Q*_*UA*_(0) = 0, *R*_*EC*_(0) = 0, *β* = 0.7, *α* = 0.66, *γ* = 0.9, *δ* = 0.8, *n*_1_ = 0.01, *n*_2_ = 0.01, *n*_3_ = 0.01, *n*_4_ = 0.02, *φ*_1_ = 0.001, *φ*_2_ = 0.005, *φ*_3_ = 0.004, *ω*_1_ = 0.0001 and *ω*_2_ = 0.0001.

**Fig 7:**
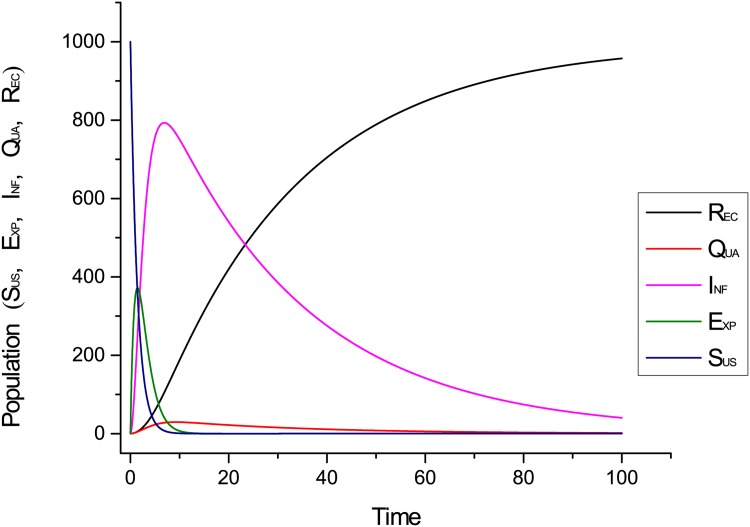
Dynamical behaviour of the proposed system.

Fig 7 shows dynamic behaviour of the proposed model with implementation of the quarantine strategy. It shows the behaviour of all states of the proposed system with respect to time. The results predict that the proposed system is asymptotically stable. In addition, It also shows that recovered class has a powerful impact on all other classes of the network. Initially, it can be seen clearly that infection is of a lesser degree but with time it increases gradually. We observe over time that *R*_*EC*_(*t*) increases whereas *I*_*NF*_(*t*) decreases. We also notice that we still have some infected nodes at 100th time unit, which proves our assumption that real network can never be completely free from infection. It also shows the important role of the quarantine strategy. The quarantined computers are kept under observation and treated with anti-virus software.

**Fig 8:**
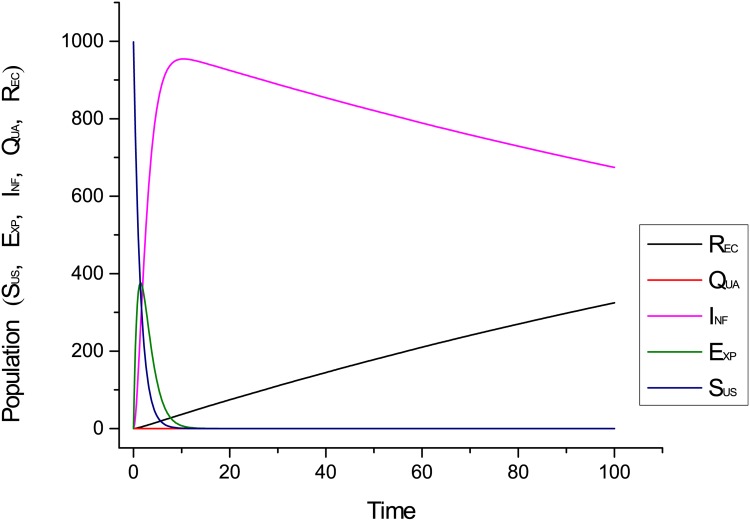
Dynamical behaviour of the proposed system without Quarantine.

Fig 8 shows the behaviour of the proposed model without implementation of any containment strategy. We see in Fig 8 that infectious hosts are diminishing very slowly and recovery process is slow as well. Fig 7 shows the behaviour of the model’s entities of the proposed system using quarantine strategy. We see from Fig 7 that infectious hosts are diminishing sharply when we applied quarantine strategy.

**Fig 9:**
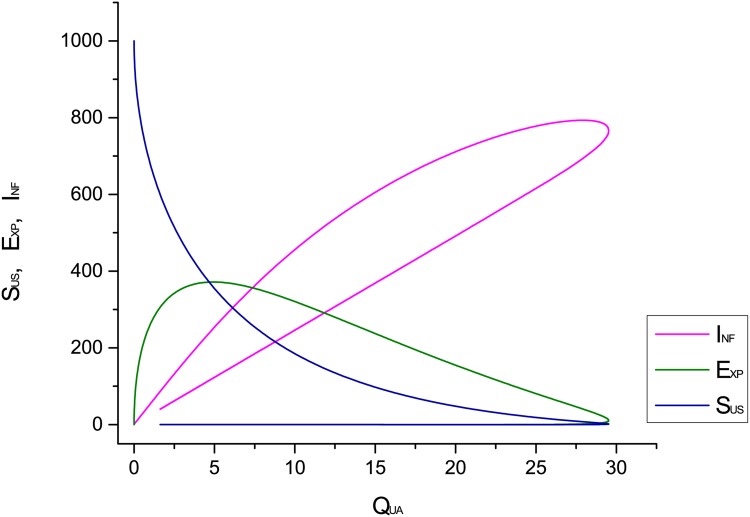
Quarantine effect on different compartments.

Fig 9 shows the relationship among quarantine, susceptible, exposed and infectious compartments. Fig 9 shows the results of susceptible, exposed and infectious classes with respect to the quarantine class. We observe in this figure that nodes from these classes (Susceptible, Exposed and Infectious) are recovering quickly with application of the quarantine strategy.

**Fig 10:**
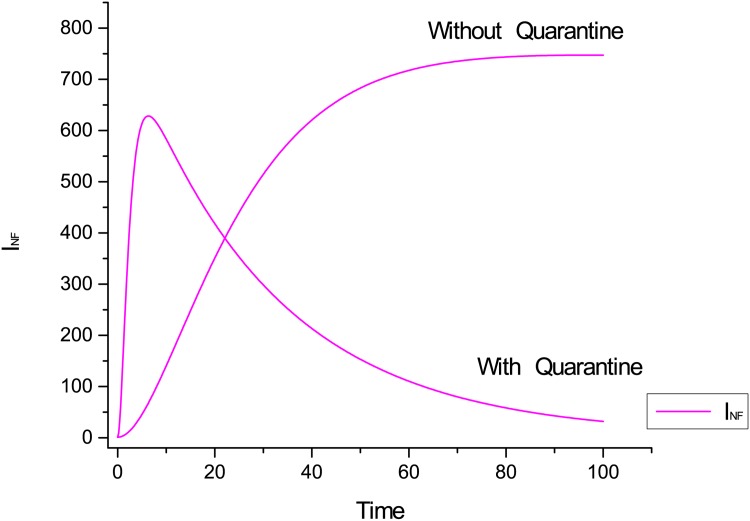
Dynamic behaviour of infectious class with and without quarantine.

Fig 10 shows the impact of quarantine over infectious compartment. Fig 10 shows the behaviour of infectious class with and without quarantine implementation. We observe that when hosts are infected then quarantine is very effective solution.

**Fig 11:**
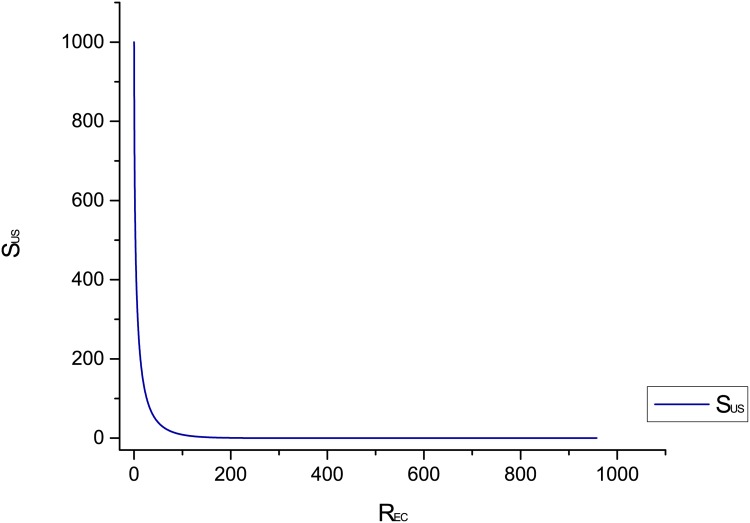
Behaviour of susceptible versus recovered compartment.

Fig 11 shows the behaviour of susceptible compartment with respect to recovered compartment. Fig 11 shows the relationship between recovered and susceptible compartments. We can observe decrease in susceptible nodes when recovered nodes are increasing. It shows that as time passes, recovered hosts increases gradually. It means susceptibility towards worm decreases with time.

For next analysis, all parameters are same except these: *γ* = 0.01, *δ* = 0.1, *n*_1_ = 0.001, *n*_2_ = 0.001, *n*_3_ = 0.001 *and* *n*_4_ = 0.002.

**Fig 12:**
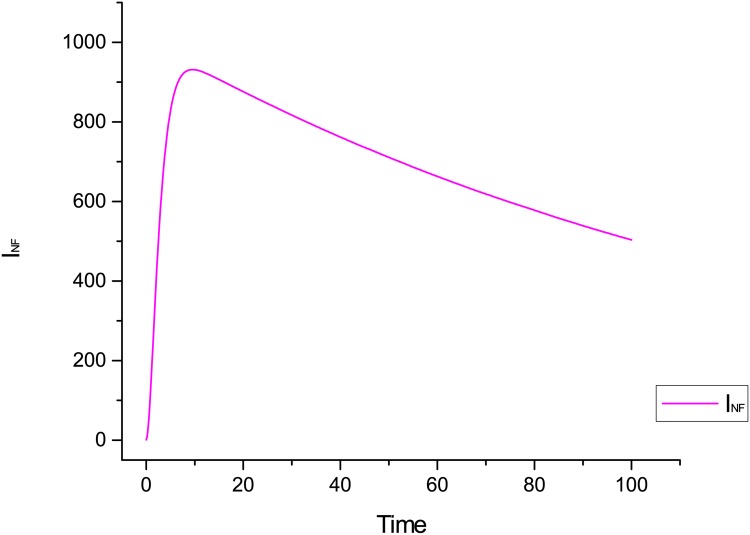
Behaviour of infectious compartment when infection rate is greater than the recovery rate.

Fig 12 shows the impact of infection rate on the performance of the proposed system. We see in Fig 12 that the worm is spreading quickly in the whole network and nodes are becoming infectious with high pace when infection rate is higher than the recovery rate.

### 3.2 Verification Results

This section contains the verification results of the proposed system. In the context of several applications, formal methods have been used to understand and characterize the behaviour of population models. *Probabilistic Model Checking* is a fully automated formal method for verifying quantitative properties of systems that exhibit stochastic behaviour. It is based on the exhaustive searching of the state space. We can check various behavioural properties regarding time and probabilities through *Probabilistic Model Checking* [44].

For quantitative analysis, we have developed a *CTMC* in *PRISM* model checker. The logic used is *Continuous Stochastic Logic (CSL)* which is an extension of Probabilistic Computation Tree Logic (PCTL). PCTL itself is a variant of CTL (Computation Tree Logic) where the path quantifiers (*A* and *E*) are replaced by a probabilistic operator (*P*) [44]. We can also use the keyword “filter” to customize the *PRISM* properties. Filters are represented using the following form:

*filter*(*op*, *prop*), where *op* denotes the operation we want to perform and *prop* denotes the property we want to verify [48].

For analysis purpose, the parameters in the experiments are defined as: *N* = 10, *S*_*US*_(0) = 9, *E*_*XP*_(0) = 0, *I*_*NF*_(0) = 1, *D*_*EL*_(0) = 0, *Q*_*UA*_(0) = 0, *R*_*EC*_(0) = 0, *β* = 0.7, *α* = 0.66, *γ* = 0.9, *δ* = 0.8, *n*_1_ = 0.01, *n*_2_ = 0.01, *n*_3_ = 0.02, *n*_4_ = 0.03, *φ*_1_ = 0.001, *φ*_2_ = 0.005, *φ*_3_ = 0.004, *ω*_1_ = 0.001 and *ω*_2_ = 0.001.

We have checked a list of behavioural properties in *PRISM* and some of these are illustrated below:

- *P* = ? [*F* *I*_*NF*_ = 0]
  This formula inquires, “what is the probability that infection will be eradicated eventually?”. This property is verified with probability 1. It means that retreat of the infection is unavoidable. The probability of reaching the state where infected individuals are 0s is 1.
- *P* = ? [*F* *R*_*EC*_ > *S*_*US*_]
  This represents the probability that recovered individuals will be greater than susceptible individuals. In other words, it means that most of the population is infected at certain points of time. This property is verified with probability 1 in the proposed model which means that most of the population first became infected at certain times and then eventually recovered.
- *P* = ? [*F* ≤ 10 *I*_*NF*_ = *N*/2]
  It shows the probability that half of the population will be infected within 10 time units. The results shows that there is a 99 percent chance of infection spreading in half of the population within 10 time units.
- *P* = ? [*F* ≤ 10 *I*_*NF*_ ≥ *S*_*US*_]
  This property inquires, “what is the probability that infected nodes will exceed the susceptible nodes within 10 time units?”. This property shows how the worm is spreading in the initial period of time. There is a 99 percent chance that infected individuals will exceed the susceptible within 10 time units.
- *P* = ? [*F* ≤ 10 *I*_*NF*_ = *N*]
  It represents the probability that the whole network will be infected within 10 time units. There is only a 12 percent chance of the infection spreading in every computer within this time.
- *P* ≥ 1 [*G* (*S*_*US*_ + *E*_*XP*_ + *I*_*NF*_ + *D*_*EL*_ + *Q*_*UA*_ + *R*_*EC*_) = *N*]
  This property represents the very important principle “Invariance”. It represents the probability that sum of all nodes will be equal to the size of the population. This should be the case in our proposed model because the population can never be negative. Since our model is based on closed population, this property should globally hold and the result shows that it is true in the proposed model.
- *R* = ? [*F* *I*_*NF*_ = 0]
  This property inquires, “what is the expected time for a network to be eventually infection free?”. The expected time for the extinction of the worm is 58 time units.
- *filter*(*forall*, *P* ≥ 1 [*F* *R*_*EC*_ = *N*])
  This states that we will eventually reach a state, initiating from any reachable state, where all hosts are recovered with probability 1. This property is true in the proposed model.
- *S* = ? [*I*_*NF*_ = 0]
  This property represents the long run probability (steady state) of the worm’s extinction from the network. The result obtained through this property shows that there is a 70 percent chance of the worm being neutralized. This shows that network is never going to be infection free, there will always be a chance of re-infection. Model checking results successfully validates the simulation results.

These behavioural properties allow us to know if behaviour of *SEIDQR(S/I)* model is stable and valid at every instance of time.

#### 3.2.1 Verification of Hybrid Quarantine Strategy using PRISM

In order to analyze the effectiveness of the quarantine strategy through *Model Checking*, the parameters in the experiments are same as defined in the above section.

Table 2 summarises the results of *Model Checking* with and without quarantine strategy based on these parameters. Model checking validates the use of quarantine strategy in worm containment. We have compared results in three scenarios. In the first scenario, we have checked the probability of the whole network becoming infectious within 10 time units. It shows a huge difference in results with and without quarantine implementation. When we have implemented quarantine strategy it shows that there is only a 12 percent chance of spreading the worm in the whole population within 10 time units but without quarantine it spreads rapidly. Without applying the quarantine strategy there is a 74 percent chance of the infection spreading throughout the whole population within 10 time units. In second scenario, we have inquired, “what are the chances that half of the population will be infected within 10 time units?”. In that particular case probability is equal in both cases with and without quarantine strategy. In third scenario, we have investigated the chances of the network becoming infection free within 100 time units. In case of the quarantine strategy, there is a 91 percent chance that whole network will be infection free within the specified time limit. In case of without the quarantine strategy, there is a 0 percent chance that whole network will be infection free within 100 time units. Table 3 summarises the results of *Model Checking* with and without quarantine strategy based on the above mentioned parameters except that population size is 26 now, susceptible hosts are 25 and infectious host is 1. In the first study, we have checked the probability of the worm infecting the whole population within 10 time units. The probability of the infection spreading across the whole population after implementing the quarantine strategy was minimal, on the contrary, without the use of the quarantine strategy the probability of infection increased by 40 percent. In the second experiment, we discovered that the probability of the infection spreading to half of the population was 0.9999 within 10 time units, with or without the implementation of the quarantine strategy. In the third experiment, we have discovered that with the implementation of the quarantine strategy, almost 69 percent of the population was infection free within 100 time units but the percentage was considerably less without quarantine. This analysis indicates the significance of implementing mitigation techniques in the initial stages of an infection which otherwise would be incredibly difficult to control and may cause severe harm to the majority of the hosts and this will be very costly. The results in Tables 2 and 3 shows the importance of implementing the hybrid quarantine strategy. It also enlightens the fact that within a larger population the speed of a worm’s spread gradually decelerates with time. The results reported in Tables 2 and 3 also indicates that smaller population recovered quickly as compared to the larger population.

**Table 2:** Results with and without Quarantine for sample size 10.

Property	Probabilities With Quarantine	Probabilities Without Quarantine
*P* = ? [*F* ≤ 10 *I*_*NF*_ = *N*]	0.1207	0.7412
*P* = ? [*F* ≤ 10 *I*_*NF*_ = *N*/2]	0.9999	0.9999
*P* = ? [*F* ≤ 100 *I*_*NF*_ = 0]	0.9131	0.0029

**Table 3:** Results with and without Quarantine for sample size 26.

Property	Probabilities With Quarantine	Probabilities Without Quarantine
*P* = ? [*F* ≤ 10 *I*_*NF*_ = *N*]	0.0013	0.3972
*P* = ? [*F* ≤ 10 *I*_*NF*_ = *N*/2]	0.9999	0.9999
*P* = ? [*F* ≤ 100 *I*_*NF*_ = 0]	0.6849	0.0016

#### 3.2.2 Verification of Qualitative Properties using Charlie

**Fig 13:**
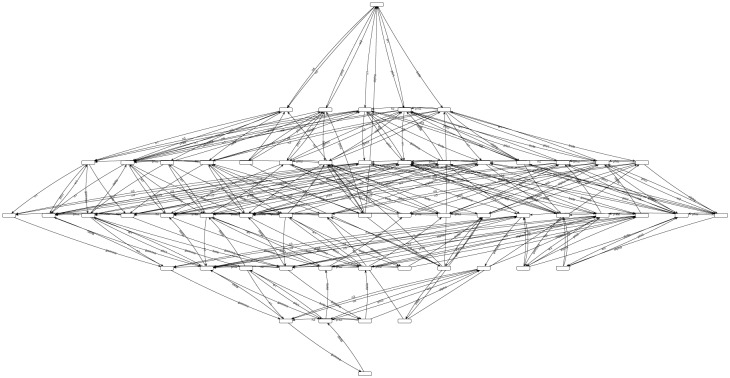
Reachability Graph. Fig 13 shows the reachability graph consisting of a total of 56 unique markings and 273 transitions with initial marking *M*_0_ = (2, 0, 1, 0, 0, 0).

The reachability graph is strongly connected which means that the graph is homogeneous. Each pair of markings are reachable from one another. Since the proposed system (compartmental model) is homogeneous therefore, the reachability graph is homogeneous. This reachability graph indicates that all markings of the model always end up in a cycle which also states that the system is deadlock free, reversible and live. This model ensures the invariance principle which means that population will never be negative. This property can be ensured by P-invariant and is verifiable in our model. Fig 13 shows the reachability graph of the *SPN*.

## 4 Discussion

In this work we have proposed a new model called *SEIDQR(S/I)* model which was based on the delays experienced by the hosts before worm containment and possible reinfection probability. We presented modelling of a compartmental model via *Petri Net* approach. Moreover, in order to study the dynamic behaviour of the proposed model we used two types of analysis techniques: *Simulation* and *Probabilistic Model Checking*.

It is important to model and study the behaviour of the system before resulting to deployment. Sometimes, models are developed after deployment as well in order to study the dynamics of the systems. The domain of communication systems are full of queries regarding cost and efficiency etc.. In order to resolve these problems it requires the construction and study of analytical and simulation models before the development and deployment of the systems. It is necessary to develop models for qualitative and quantitative understanding of the systems. The nature of traffic between communication systems is unpredictable and therefore, it is typical to develop a stochastic model to represent such systems [39]. Therefore, in this study we have used *SPN* to construct models. Mathematical models have been conventionally used for analysis of worm propagation. However, these models rely on unreasonable assumptions [39] and cannot be formally verified. In this work, we have used the *SPN* to model worm propagation. Then, *CTMC* of the proposed model is constructed in *PRISM* model checker.

Our results are comparable to Mishra and Tyagi [49] where dynamical behaviour is achieved after quarantining 50 nodes. Our model encapsulated a population of 1000 nodes and generated results more accurately by quarantining 30 nodes while retaining similar behavioural curves, as that of Mishra and Tyagi [49].

The analysis of the *CTMC* strongly validates the results obtained through simulation of the *SPN*, which makes the proposed framework valid for application in the field of epidemiology. The analysis obtained through *Model Checking* also supports assumption that real network is never infection free. There is always a chance of possible re-infection. We have already showed this through simulation of the *SPN*. We verified the structural property of the *SPN* through *Charlie* tool. Since the proposed model is homogeneous, it should be strongly connected and this was confirmed through *Charlie* tool. We have also verified some behavioural properties of the *SPN* such as reachability, liveliness, reversibility and deadlock freeness through *Charlie* tool.

A major disadvantage of the modelling approach is the computational cost of the method. In order to evaluate quantitative properties via the *Probabilistic Model Checking* method, it requires a reasonable amount of time (in hours). To overcome this computational obstacle we had to limit our sample size to the maximum of 26 hosts. A possible solution to this problem is to use *Approximate Model Checking* [50]. However, *Probabilistic Model Checking* offers promising outcomes in the analysis of dynamics of compartmental models and therefore, it is worth further investigation.

## 5 Conclusion and Future Work

In this paper, we have proposed a framework to formally verify and validate the compartmental models via model checking and simulation. The proposed framework can be applied to any epidemiological compartmental network model. Beginning with the development of a *SEIDQR(S/I)* model in *Petri Nets* and *PRISM*, we were able to get insight into how *SEIDQR(S/I*) works. On the basis of proposed methodology, we were able to simulate as well as investigate the *SEIDQR(S/I)* model through queries encoded in *CSL*. By varying different parameters of the proposed model, we verified its behavioural properties. Using this approach, we checked certain situations through these properties such as when the worm’s infection will be at its peak point, its duration and so on and so forth. The *Petri Net* approach described here has allowed us to perform modelling of the system easily and quickly as compared to other analysis methods. According to this work, we have come to the conclusion that quarantine strategies are extremely effective in reducing the risk of propagating the worm and, in fact, have an outstanding effect in regulating it. *Probabilistic model checking* allowed us to explore many behavioural properties of our model. The proposed approach is applicable for both understanding worm propagation and developing more challenging worm defence strategies. There are many factors that effect worm propagation such as delay, bandwidth and activity of device in the network, which cannot be neglected and will be taken into consideration in our future works. We will use Entropy based measure to compute different properties of the proposed model and will counter verify it with *Probablistic Model Checking Approach* in the futrue work. Since negative exponential distribution is used to generate stochastic transitions, sensitivity analysis of the proposed model will be performed in the next study.

## Supporting Information

S1 Table (PDF)

S1 File (PDF)

S2 File (PDF)
