# Supplementary material for: Petri Net and Probabilistic Model Checking Based Approach for the Modelling, Simulation and Verification of Internet Worm Propagation
Source: PLoS One. 2015 Dec 29;10(12):e0145690. doi: 10.1371/journal.pone.0145690 (PMC4699213; doi:10.1371/journal.pone.0145690)
Supplement: S1 Table — (PDF) [file pone.0145690.s001.pdf]

List of Abbreviations

These are the abbreviations used throughout the paper.

**Term**	**Abbreviation**
SPN	Stochastic Petri Net
CTMC	Continuous Time Markov Chain
SEIR	Susceptible-Exposed-Infectious-Recovered
SEIDQR(S/I)	Susceptible-Exposed-Infectious-Delayed-Recovered (Susceptible/Infectious)
CSL	Continuous Stochastic Logic
CTL	Computation Tree Logic
PCTL	Probabilistic Computation Tree Logic
SEM	Simple Epidemic Model
TFM	Two Factor Model
AAWP	Analytical Active Worm Propagation
KM	Kermack-Mckendrick
LAAWP	Local Analytical Active Worm Propagation
