# Supplementary material for: Petri Net and Probabilistic Model Checking Based Approach for the Modelling, Simulation and Verification of Internet Worm Propagation
Source: PLoS One. 2015 Dec 29;10(12):e0145690. doi: 10.1371/journal.pone.0145690 (PMC4699213; doi:10.1371/journal.pone.0145690)
Supplement: S1 File — (PDF) [file pone.0145690.s002.pdf]

```
ctmc                        //Specifying the type of probabilistic
                            model

const int Max;              //Upper limit
const int N;                //Population size
const double v_beta;        //The rate at which susceptible hosts
                            become exposed
const double v_alpha;       //The rate at which exposed hosts
                            become infectious
const double v_n1;          //Delayed rate of susceptible hosts
const double v_n2;          //Delayed rate of Exposed hosts
const double v_n3;          //Delayed rate of Infectious hosts with
                            anomaly detection
const double v_n4;          //Delayed rate of infectious hosts with
                            signature based detection
const double v_phi1;        //The rate at which susceptible hosts
                            become recovered
const double v_phi2;        //The rate at which exposed hosts
                            become recovered
const double v_phi3;        //The rate at which infectious hosts
                            become recovered
const double v_omega1;      //The rate at which recovered hosts
                            become susceptible
const double v_omega2;      //The rate at which recovered hosts
                            become infectious
const double v_gamma;       //Quarantined rate of delayed hosts
const double v_delta;       //Recovery rate of quarantined hosts

module  SEIDQRSI            //Name of the module

//Definition of State Variables
DEL:  [ 0..Max ]  init 0;     //Delayed Hosts
INF:  [ 0..Max ]  init 1;     //Infectious Hosts
QUA:  [ 0..Max ]  init 0;     //Quarantined Hosts
REC:  [ 0..Max ]  init 0;     //Recovered Hosts
```

```
Sus:  [ 0..Max ]  init N-1;    //Susceptible Hosts
Exp:  [ 0..Max ]  init 0;      //Exposed Hosts

//Definition of Transition Rules
//If guards are met then state variable will be updated by the
specified rate.
[n4]
(Inf > 0) & (Del < Max )-> (v_n4) * Inf :
(Del' = Del + 1) & (Inf' = Inf - 1);
[omega2]
(Rec > 0) & (Inf < Max )-> (v_omega2) * Rec :
(Inf' = Inf + 1) & (Rec' = Rec - 1);
[phi2]
(Exp > 0) & (Rec < Max )-> (v_phi2) * Exp :
(Exp' = Exp - 1) & (Rec' = Rec + 1);
[phi3]
(Inf > 0) & (Rec < Max )-> (v_phi3) * Inf :
(Inf' = Inf - 1) & (Rec' = Rec + 1);
[n3]
(Inf > 0) & (Del < Max )-> (v_n3) * Inf :
(Del' = Del + 1) & (Inf' = Inf - 1);
[n1]
(Sus > 0) & (Del < Max )-> (v_n1) * Sus :
(Del' = Del + 1) & (Sus' = Sus - 1);
[omega1]
(Rec > 0) & (Sus < Max )-> (v_omega1) * Rec :
(Rec' = Rec -1) & (Sus' = Sus + 1);
[phi1]
(Sus > 0) & (Rec < Max )-> (v_phi1) * Sus :
(Rec' = Rec + 1) & (Sus' = Sus - 1);
[delta]
```

```
(Qua > 0) & (Rec < Max )-> (v_delta) * Qua :
(Qua' = Qua - 1) & (Rec' = Rec + 1);
[gamma]
(Del > 0) & (Qua < Max )-> (v_gamma) * Del :
(Del' = Del - 1) & (Qua' = Qua + 1);
[n2]
(Exp > 0) & (Del < Max )-> (v_n2) * Exp :
(Del' = Del + 1) & (Exp' = Exp - 1);
[alpha]
(Exp > 0) & (Inf < Max )-> (v_alpha) * Exp :
(Exp' = Exp - 1) & (Inf' = Inf + 1);
[beta]
(Sus > 0) & (Exp < Max )-> (v_beta) * Sus :
(Exp' = Exp + 1) & (Sus' = Sus - 1);

endmodule      //end of module

//Definition of the reward
rewards "time"
 true : 1;     //assign reward 1 to each state
endrewards
//end of reward
```
