# Supplementary material for: Petri Net and Probabilistic Model Checking Based Approach for the Modelling, Simulation and Verification of Internet Worm Propagation
Source: PLoS One. 2015 Dec 29;10(12):e0145690. doi: 10.1371/journal.pone.0145690 (PMC4699213; doi:10.1371/journal.pone.0145690)
Supplement: S2 File — (PDF) [file pone.0145690.s003.pdf]

Software

Snoopy tool is used for Petri Net modelling and simulation of the system. PRISM model checker is used for analysis of the quantitative properties and quarantine strategy. Charlie tool is used for analysis of the qualitative properties of the system.

Snoopy

Snoopy is a tool used to design, animate and simulate Petri Nets. Snoopy is comprised of many frameworks like stochastic, hybrid and continuous Petri Nets. It also allows construction of high level Petri Nets like colored Petri Nets. This tool is used for formal verification and validation of systems from different domains. It allows us to investigate a Petri Net in various complementary ways. The SPN of the proposed model is constructed in Snoopy tool.

PRISM

PRISM is a probabilistic model checker and is used to automatically verify probabilistic systems using continuous stochastic logic. It provides support for three types of probabilistic models: (1) DTMC (2) MDP and (3) CTMC. PRISM accepts the probabilistic model written in the PRISM modelling language, builds the corresponding probabilistic model against it and then uses CSL for the verification of the behavioural properties of the model. This research work focuses on the CTMC developement of the proposed model in PRISM model checker and verification of the properties encoded in CSL.

Charlie

Charlie is a software tool that performs analysis of Petri Nets. It is used to verify the list of properties of the Petri Nets such as reachability, liveness and boundedness etc. This tool is used for the verification as well as validation of the systems in different domains. Charlie is used to perform structural, invariants based, reachability graph based and behavioural analysis of the Petri Nets. In this paper, we have used Charlie to perform qualitative analysis of different properties of the proposed system.
